# Comparison of statistical models for estimating intervention effects based on time-to-recurrent-event in stepped wedge cluster randomized trial using open cohort design

**DOI:** 10.1186/s12874-022-01552-6

**Published:** 2022-04-26

**Authors:** Shunsuke Oyamada, Shih-Wei Chiu, Takuhiro Yamaguchi

**Affiliations:** 1 Division of Biostatistics, Tohoku University Graduate School of Medicine, Sendai, Japan; 2 Departments of Biostatistics, JORTC Data Center, Tokyo, Japan

**Keywords:** Stepped-wedge, Cluster randomized trial, Open cohort design, Recurrent event, Time-to-event, Statistical model, Time-dependent covariate, Simulation, Comparison

## Abstract

**Background:**

There are currently no methodological studies on the performance of the statistical models for estimating intervention effects based on the time-to-recurrent-event (TTRE) in stepped wedge cluster randomised trial (SWCRT) using an open cohort design. This study aims to address this by evaluating the performance of these statistical models using an open cohort design with the Monte Carlo simulation in various settings and their application using an actual example.

**Methods:**

Using Monte Carlo simulations, we evaluated the performance of the existing extended Cox proportional hazard models, i.e., the Andersen-Gill (AG), Prentice-Williams-Peterson Total-Time (PWP-TT), and Prentice-Williams-Peterson Gap-time (PWP-GT) models, using the settings of several event generation models and true intervention effects, with and without stratification by clusters. Unidirectional switching in SWCRT was represented using time-dependent covariates.

**Results:**

Using Monte Carlo simulations with the various described settings, in situations where inter-individual variability do not exist, the PWP-GT model with stratification by clusters showed the best performance in most settings and reasonable performance in the others. The only situation in which the performance of the PWP-TT model with stratification by clusters was not inferior to that of the PWP-GT model with stratification by clusters was when there was a certain amount of follow-up period, and the timing of the trial entry was random within the trial period, including the follow-up period. In situations where inter-individual variability existed, the PWP-GT model consistently underperformed compared to the PWP-TT model. The AG model performed well only in a specific setting. By analysing actual examples, it was found that almost all the statistical models suggested that the risk of events during the intervention condition may be somewhat higher than in the control, although the difference was not statistically significant.

**Conclusions:**

When estimating the TTRE-based intervention effects of SWCRT in various settings using an open cohort design, the PWP-GT model with stratification by clusters performed most reasonably in situations where inter-individual variability was not present. However, if inter-individual variability was present, the PWP-TT model with stratification by clusters performed best.

**Supplementary Information:**

The online version contains supplementary material available at 10.1186/s12874-022-01552-6.

## Background

A cluster randomised trial (CRT) is a randomised trial design in which a cluster of regions or sites is used when it is not possible or appropriate to assign an intervention to an individual patient, like a randomised controlled trial (RCT) [1, 2]. The stepped wedge CRT (SWCRT) is a type of CRT, in which multiple randomization procedures are enforced temporally to switch into intervention, and all clusters are sequentially transferred (unidirectional switch) from the control condition to the intervention condition [3, 4]. For the sake of simplicity, it is assumed that the intervention effect will persist from the time the control condition is switched to the intervention condition until the end of the trial.

There are three main types of SWCRT design: (i) continuous recruitment short exposure, (ii) closed cohort, and (iii) open cohort [5]. In the open cohort design, each subject is assessed repeatedly at a series of measurement points or at a subject-specific time point, such as the occurrence of an event. In this design, subjects may get enrolled or censored the trial at any time during the trial period based on pre-specified eligibility criteria. Thus, some subjects are exposed to both control and intervention conditions during the trial, while others are only exposed to one.

**Fig. 1:**
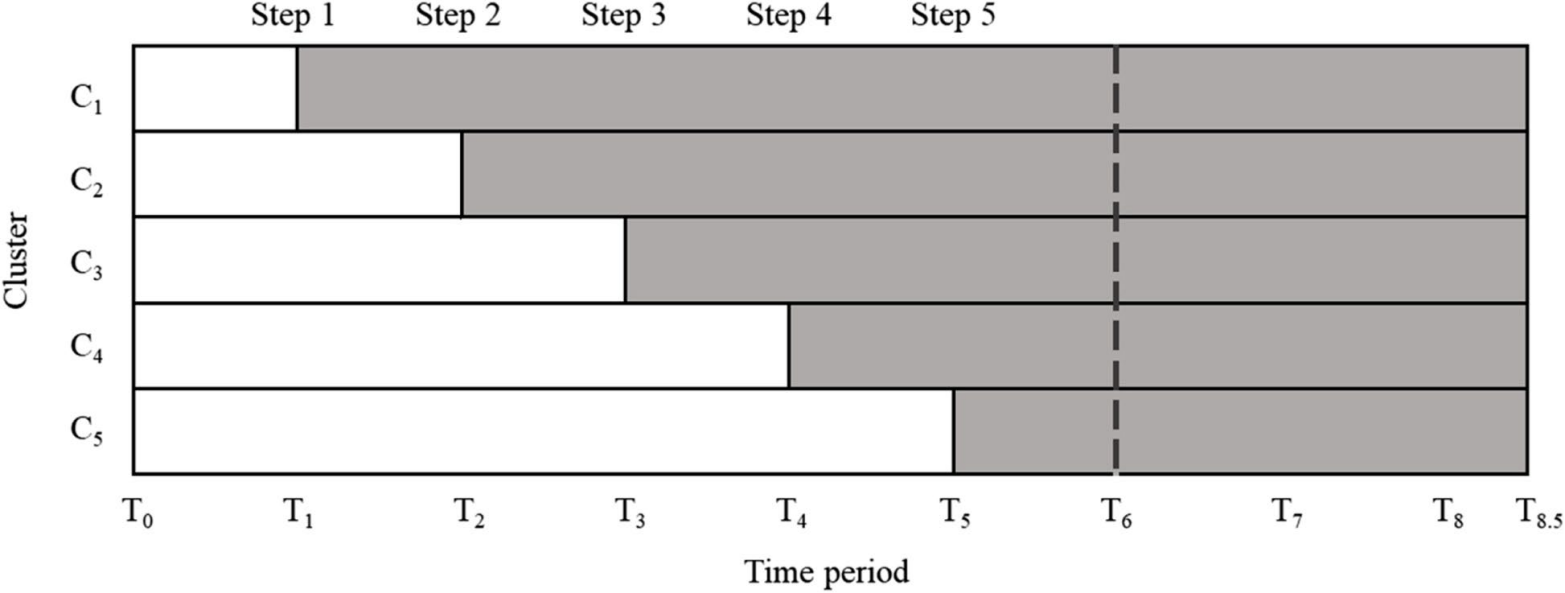
Schematic representation of the actual example. White cells correspond to the periods during which the residents received the standard-of-care (control condition), and grey cells correspond to periods during which the residents received new interventions (intervention condition). Each cluster ${C}_{1}$ to ${C}_{5}$ contains two or three facilities, and one cluster moves from the control condition to the intervention condition in Steps 1 to 5. The duration for one Step (between one Step and the next) is two months, and the time period ${T}_{0}$ to ${T}_{8}$ is also every two months. The duration between ${T}_{8}$ and ${T}_{8.5}$ is one month. The start of the trial is ${T}_{0}$, and after ${T}_{6}$, which is the end of the last step period, there is a follow-up period for 5 months until ${T}_{8.5}$ (equivalent to 2.5 Steps)

The INSPIRED trial, which is the actual example used in this study, was a multi-centre SWCRT that examines whether a model of care that provides specialist palliative care interventions in residential care homes (i.e. the intervention condition) leads to fewer (acute care) hospitalisations and shorter lengths of stay in hospital for care home residents, when compared to usual care (i.e. the control condition) [6]. A schematic representation of actual example is presented in Fig. 1. It is an open cohort design as all the residents in each facility at the start of the trial and all-new enrolments to the facility after the start of the trial were included. Many residents were exposed to both the control and intervention conditions, as they remained in their residences continuously unless they died or were discharged from the care home. The primary outcome was the length of the hospital stays, and the secondary outcomes were the number of hospitalisations and the cost.

Some residents never experienced hospitalisation, while others were repeatedly hospitalised in the actual example. The same event repeatedly occurs over time to the same individual, such as the hospitalisation in the actual example, is called a recurrent event [7]. A common way to analyse recurrent event is the recurrence rate (average number of recurrences per unit time), which corresponds to the number of hospitalisations per facility-month as a secondary outcome in the actual example. This analysis requires the assumption that the incidence of hospitalisation is always constant in the interval per facility-month which is generally a strong assumption. In addition, even if the number of hospitalisations per facility-month are the same, there may be differences in the time it takes for each hospitalization to occur, and this is called the time to hospital admission (TTHA) and may represent the effects of the intervention. Since admission and discharge data are collected for each hospitalisation in the actual example, that is, the TTHA is measured repeatedly, it may be useful to evaluate hospitalisations as recurrent events within the framework of a time-to-event (TTE) analysis.

When assessing the impact of a covariate on the TTE with a hazard ratio (HR), the Cox proportional hazard (CoxPH) model is most often used [8], and it assumes that the event is a one-time terminal event. When the CoxPH model is applied to recurrent events, only time-to-first-event (TTFE) can be included in the analysis. Against this background, the extension of the CoxPH model to recurrent events has been actively pursued, especially in the 1980s [9–11], and it has mainly been used to evaluate the time-to-recurrent-event (TTRE) in RCTs.

Methods to analyse TTE in SWCRT are currently unclear [12]. SWCRT using an open cohort design, by its nature, must deal with subjects who are exposed to both the control and intervention conditions (observed across the unidirectional switch). When estimating the intervention effects based on the TTFE, if the change in the time-dependent covariate is independent of TTE, then the unidirectional switch in the CoxPH model can be explained using the time-dependent covariate [13, 14], and methodological studies on the performance in the context of SWCRT have previously been conducted [15]. In TTRE, the existing extended CoxPH model with time-dependent covariates possibly apply to SWCRT with unidirectional switching [16–18]. In addition, CRT is known to have a problem with cluster effects when the outcomes of individuals in the same cluster become similar for various reasons. In other words, it means that there will be an increase in the variability of regression coefficients among clusters, which is also a concern in SWCRT. When estimating intervention effects based on TTFE, the cluster effect in SWCRT can be treated using the CoxPH model stratified by clusters [15], as it assumes that each cluster’s baseline hazard function is different. For TTRE, the existing extended CoxPH model stratified by clusters possibly be used.

To our knowledge, there are currently no methodological studies on the performance of the statistical models for estimating intervention effects based on TTRE in SWCRT with an open cohort design, or examples of its application to actual studies. Investigating the performance of the statistical models used to estimate intervention effects based on TTRE in SWCRT using an open cohort design in various settings, may contribute to the selection of statistical models for the actual planning and analysis of SWCRT.

The purpose of this study was to evaluate “which statistical models resulted in better performance estimating intervention effects using TTRE in SWCRT with an open cohort design” with the Monte Carlo simulation (hereafter, simulation) in various settings. We also applied each statistical model to hospital admission data to test the actual example and interpreted the results based on the simulation results.

## Methods

### Actual example

Details of the trial design, interventions, resident background information, and efficacy results of the INSPIRED trial have been published previously [6]. The trial included 1700 residents from 12 care homes in Australia, of which 1089 (64.1%) were residents at the start of the trial, and the remaining 611 (35.9%) became residents after the start of the trial. There were 1149 hospitalisations during the trial, of which 943 hospitalizations of more than 24 h (> 24 h) were used for the primary outcome, length of stay in hospital. Of the residents, 377 had only one hospitalization of > 24 h, while 211 had multiple hospitalizations of > 24 h (137 had two, 45 had three, 11 had four, and 18 had four or more). The number of residents who died during the trial period was 534 (31.4%). The secondary outcome, number of hospitalizations > 24 h per facility-month, was 5.6 in the control condition and 4.3 in the intervention condition, a decrease of approximately 23% (no adjustment by covariates or comparison by estimation/statistical testing was performed).

### Basic notation

The timing of the unidirectional switch (henceforth, switch) in each cluster of the SWCRT is called a step, and here, we consider SWCRT with $m$ clusters and $s$ steps. For simplicity, we assume that the number of clusters to be switched from the control condition to the intervention condition in one step is one ($s=m$). In the $i$ th cluster ($i=1,\:\dots ,\:m$), ${n}_{i}$ is the number of subjects observed during the entire trial duration.

Assuming that the start of the test is ${t}_{S}$ and the end of the last step period is ${t}_{E}$, the timing of the switch in each cluster is calculated as follows: ${W}_{i}={t}_{S}+i*({t}_{E}-{t}_{S}) / (m+1)$, and the distance between switches is calculated as follows: ${W}_{d}={W}_{i+1}-{W}_{i}={W}_{i}-{W}_{i-1}=({t}_{E}-{t}_{S}) / (m+1)$. Let ${d}_{ij}$ be the time point at which the $j$ th subject ($j=1,\: \dots ,\: {n}_{i}$) in the $i$ th cluster entered the trial. The distance ${w}_{ij}$ to the switch for each subject from the trial entry is defined as follows:

$$\left\{\begin{array}{cc}{w}_{ij}={W}_{i}-{d}_{ij}& { W}_{i}\ge {d}_{ij}\\ {w}_{ij}=0& {W}_{i}<{d}_{ij}\end{array}\right.$$

Suppose the starting point of the second and subsequent TTREs is the time of the previous event, and the actual TTRE used in the analysis based on the kth recurrence is ${T}_{ijk}$. Considering the starting point of each recurrence, the distance ${w}_{ijk}$ to the switch for each subject is defined as follows:

$$\left\{\begin{array}{cc}{w}_{ijk}={W}_{i}-{d}_{ij} & { W}_{i}\ge {d}_{ij} \:(k=1)\\ {w}_{ijk}={W}_{i}-{T}_{ijk-1}& { W}_{i}\ge {T}_{ijk-1} \:(k\ge 2)\\ {w}_{ijk}=0& {W}_{i}<{d}_{ij} \: \left(k=1\right), { \:W}_{i}\ge {T}_{ijk-1} \:(k\ge 2)\end{array}\right.$$

where ${h}_{ijk}\left(t\right)$ is the hazard function of the $k$ th recurrence of the $j$ th subject in the $i$ th cluster at time $t$, and ${h}_{0ik}\left(t\right)$ is the baseline hazard function of the $k$ th recurrence of the $i$ th cluster at time $t$. No specific distribution is assumed for the baseline hazard function. ${Y}_{ijk}\left(t\right)$ is the indicator variable for the $k$ th recurrence of the $j$ th subject in the $i$ th cluster at time $t$, and this is 1 if the subject is at risk of recurrence and under observation, and 0 if not. ${X}_{ijk}$ is a vector of time-independent covariates for the $k$ th recurrence of the $j$ th subject in the $i$ th cluster, and ${\beta }_{ik}$ is a vector of fixed parameters for the time-independent covariates of the $k$ th recurrence of the $i$ th cluster. ${Z}_{ijk}\left(t\right)$ is the intervention indicator as a time-dependent covariate for the $k$ th recurrence of the $j$ th subject in the $i$ th cluster, which is 0 for ${t<{w}_{ij} \mathrm{\:or\: }w}_{ijk},$ and 1 for ${t\ge {w}_{ij} \mathrm{\:or\:} w}_{ijk}$ (changes before and after the switch). ${\beta }_{tik}$ is the parameter for the intervention effect for the $k$ th recurrence of the $i$ th cluster. The subscript $i$ is omitted if it is assumed that each cluster has a common effect. The subscript $k$ is omitted if it is assumed that each recurrence has a common effect.

### Statistical models

The first model considered was the CoxPH model [8, 14]. The hazard of the $j$ th subject in the $i$ th cluster at time $t$ is expressed as follows:.

$${h}_{ij}\left(t\right)={h}_{0i}\left(t\right) \mathrm{exp}({\beta }_{ti}{Z}_{ij}\left(t\right)+{\beta }_{i}^{^{\prime}}{X}_{ij})$$

As was previously mentioned, applying the CoxPH model to recurrent events would result in a loss of information because only the TTFE of each subject can be included in the analysis, and the second and subsequent events are ignored. Taking recurrent events into account should theoretically improve the efficiency of estimating the effects of interventions [19]. Since the purpose of this study is to evaluate the performance of the statistical model in estimating the intervention effect using TTRE, no performance evaluation on the CoxPH model will be conducted. In the following, we present an extended CoxPH model that allows for the inclusion of TTRE in the analysis.

The Andersen and Gill (AG) model assumes a common baseline hazard function for all events, independent of the number of previous recurrences, and it is considered beneficial when investigating the overall intervention effect on the occurrence of recurrent events [9]. The hazard for the $j$ th subject in the $i$ th cluster at time $t$ is expressed as follows:

$${h}_{ij}\left(t\right)={Y}_{ij}\left(t\right) {h}_{0i}\left(t\right) \mathrm{exp}({\beta }_{ti}{Z}_{ijk}\left(t\right)+{\beta }_{i}^{^{\prime}}{X}_{ijk})$$

In the usual CoxPH model, a subject who has experienced one event is no longer at risk for that event. In contrast, the AG model assumes that subjects who have experienced at least one event remain at risk unless they drop out of the trial. In the AG model, multiple events that occur within the same subject are considered to be independent. However, because they may not be independent in reality, it is advised that robust variance is used to handle the correlation within the subject when inferring the parameter vector [20, 21].

The Prentice-Williams-Peterson (PWP) model assumes a different baseline hazard function for each recurrence and accounts for correlation by stratifying by the number of prior recurrences. Therefore, it is considered beneficial when the risk of repeat events differs between recurrences [17]. The hazard ${h}_{ijk}\left(t\right)$ for the $k$ th recurrence is defined by the history of the covariates and the number of recurrences up to time $t$. Conditionally, it is assumed that the ($k-1$)th recurrence is independent of the $k$ th recurrence. Furthermore, it assumes that the subject is not at risk for the $k$ th recurrence until the ($k-1$)th recurrence, so that ${Y}_{ijk}\left(t\right)$ is 0 until the ($k-1$)th recurrence and 1 after that.

The PWP model can be broadly divided into two models depending on the treatment of the time points. First, the PWP total-time (PWP-TT) model uses the time from the start of the follow-up to each recurrence. The hazard of the $k$ th recurrence of the $j$ th subject in the $i$ th cluster at time $t$ is expressed as follows:

$${h}_{ijk}\left(t\right)={Y}_{ijk}\left(t\right) {h}_{0ik}\left(t\right) \mathrm{exp}({\beta }_{tik}{Z}_{ijk}\left(t\right)+{\beta }_{ik}^{^{\prime}}{X}_{ijk})$$

The second is the PWP gap-time (PWP-GT) model, which uses the time from the occurrence of the previous recurrence to each recurrence. The hazard of the $k$ th recurrence for the $j$ th subject in the $i$ th cluster at time $t$ is expressed as:

$${h}_{ijk}\left(t\right)={Y}_{ijk}\left(t\right) {h}_{0ik}\left(t-{t}_{k-1}\right) \mathrm{exp}({\beta }_{tik}{Z}_{ijk}\left(t\right)+{\beta }_{ik}^{^{\prime}}{X}_{ijk})$$

As the number of recurrences increases in the PWP model, the number of subjects at risk becomes relatively small. This would make the estimates unstable, so limiting the data to a specific number of recurrences is usually necessary [22]. Due to these characteristics, the PWP model is helpful in situations where the number of recurrences per subject is small [17]. Our study assumes that each recurrence has a common effect when estimating parameters using the PWP model.

For each of the statistical models described so far, there are two analysis policies: (i) with stratification by clusters, which assumes that the baseline hazard function is different for each cluster, and (ii) without stratification by clusters, which assumes that the baseline hazard function is the same for each cluster.

The performance of each statistical model in the simulation was evaluated in terms of bias, mean square error (MSE), and coverage probability (CP). Bias is the mean difference across simulated replicates of the parameters of the intervention effect based on each statistical model and the true intervention effect ${\beta }_{t}$, where a positive value indicates underestimation and a negative value indicates overestimation; MSE is the sum of bias squared and variance of the estimated intervention effect based on each statistical model, with smaller values indicating better performance. CP is the proportion of the 95% confidence interval (CI) for the HR obtained by each statistical model that includes the HR based on the true intervention effect ${\beta }_{t}$. The closer the CI is to 0.95, the better the performance.

### Data generation process

For the time point ${d}_{ij}$ of the $j$ th subject in the $i$ th cluster to enter in the trial, we use ${t}_{S}$ at the beginning of the trial and ${t}_{E}$ at the end of the last step period already mentioned, and generate them randomly within the interval of ${t}_{S}+(\left({t}_{E}-{t}_{S}\right)*e)/E$ or ${t}_{S}+(\left({t}_{F}-{t}_{S}\right)*e)/E$. From this point, the TTFE at least, always occurs starting from ${d}_{ij}$. Here, $e$ is a pseudo-random number generated from a uniform distribution, $e\sim U(0, 1)$.

${t}_{F}$ indicates the end of the trial and is expressed as ${t}_{F}={t}_{E}+({W}_{d}*F)$ using the distance ${W}_{d}$ between ${t}_{E}$ and the switch at the end of the last step period, as described above. $F$ is a coefficient that specifies the follow-up period that may be set after the end of the last step period. When $F=0$, there is no follow-up period, and ${t}_{F}={t}_{E}$. If $F=X(>1)$, there is a follow-up period of $X$ step after the end of the last step period. In the actual example, as shown in Fig. 1, each step is set every two months, and there is a follow-up period of 5 months (= 2.5 steps) after the end of the last step period. Based on the purpose and setting of the trial, other SWCRT have adopted a similar design [23–25].

**Fig. 2:**
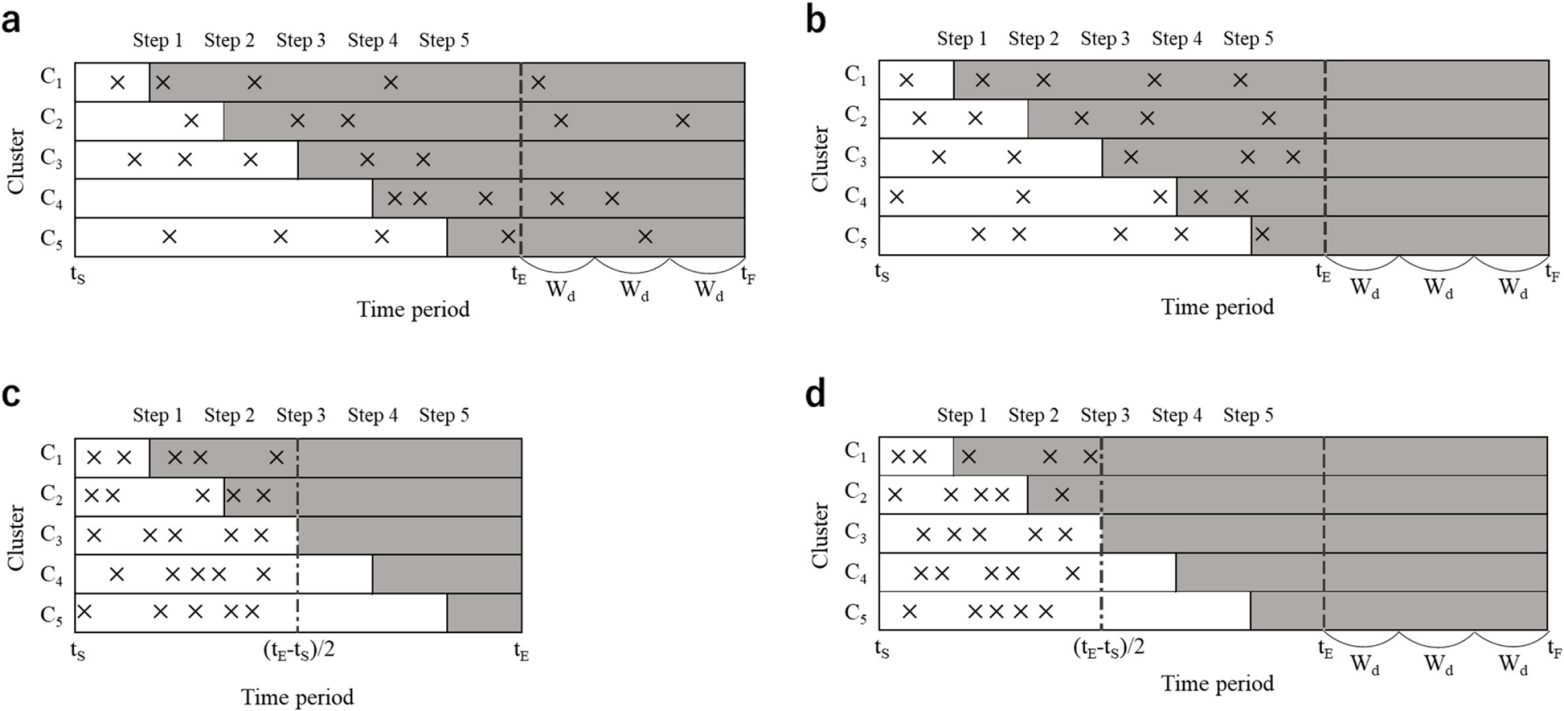
Schematic diagram of the simulation considering the follow-up period and the timing of trial entry. White cells correspond to the control condition and grey cells to the intervention condition. Cross marks show examples of the time points when the five subjects in each cluster entered the trial. $F$ is a coefficient that specifies the follow-up period that may be set after the end of the last step period. When $F=0$, there is no follow-up period, and ${t}_{F}={t}_{E}$. If $F=X(>1)$, there is a follow-up period of $X$ step after the end of the last step period. $E$ is a coefficient that specifies the timing of the trial entry. If $E=1$, the subject enters the trial randomly between ${t}_{S}$ and ${t}_{E}$ or ${t}_{F}$, which reflects the open cohort design in that the subject may enter in the trial at any time. If $E$ is greater than 1, it reflects a situation where the entry of the trial is concentrated at an earlier stage of the trial. **a** Example of a case where $F=3, E=1$, and trial entry is allowed until the follow-up period. **b** Example of setting $F=3, E=1$ and trial entry is terminated in the final step period. **c** Example of setting $F=0, E=2$. **d** Example of setting $F=3, E=2$

In the actual simulation, three policies are considered: (i) no follow-up period and ${d}_{ij}={t}_{S}+(\left({t}_{E}-{t}_{S}\right)*e)/E$; (ii) there is a follow-up period and ${d}_{ij}={t}_{S}+(\left({t}_{F}-{t}_{S}\right)*e)/E$ (allow trial entry until the end of the follow-up period; illustrated in Fig. 2a); (iii) there is a follow-up period but ${d}_{ij}={t}_{S}+(\left({t}_{E}-{t}_{S}\right)*e)/E$ (terminate trial entry at the end of the last step period; illustrated in Fig. 2b).

In addition, $E$ is a coefficient that specifies the timing of the trial entry. If $E=1$, the subject enters the trial randomly between ${t}_{S}$ and ${t}_{E}$ or ${t}_{F}$, which reflects the open cohort design in that the subject may enter in the trial at any time. If $E$ is greater than 1, it reflects a situation where the entry of the trial is concentrated at an earlier stage of the trial (illustrated in Fig. 2c). In the actual example, 64.1% of the residents entered at the start of the trial. Depending on the purpose and setting of the trial, other SWCRT show similar situations [26, 27].

In the actual simulation, policies (i) to (iii) above regarding the follow-up period and the time of trial entry can be taken for $E=1$ and $E>1$, respectively. Our study adopts only policy (iii) instead of (ii) at $E>1$ (illustrated in Fig. 2d).

To compare our results with the secondary outcome of the actual example, number of hospitalisations > 24 h per facility-month, we decided to treat only hospitalizations > 24 h as a TTE in this study. It was previously published [6] that the number of residents repeatedly hospitalised more than four times was very small. Therefore, in our study, the maximum number of recurrent events generated in the simulation was three.

The relative performance of the statistical models used in TTRE, which are based on bias and variability, depend on the event generation model used in the simulation, and it is thus recommended that simulations based on multiple event generation models be considered [28]. Therefore, in this study, three types of event generation model were used.

**Fig. 3:**
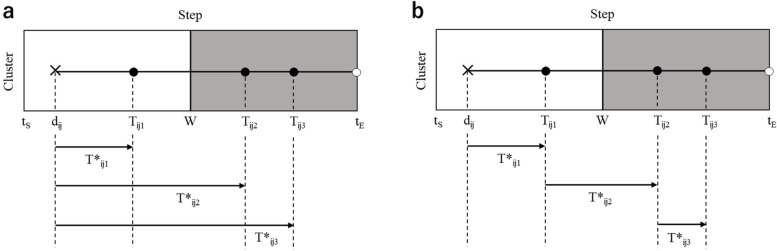
Visualization of the event generation models. White cells correspond to the control condition and grey cells to the intervention condition. Cross marks indicate when a subject enters the trial, filled black circles indicate relapse, and filled white circles indicate censoring. **a** Example of the Poisson process and the Mixed-Poisson process: all three time-to-events occur at the time of trial entry. **b** Example of the Weibull model: the first time-to-event occurs at the time of trial entry, and the second and subsequent time-to-events occur at the time of the previous event

The first is the Poisson process, which generates TTEs based on exponential distributions independent of each other, not only between subjects but also within subjects. The exponential distribution consists only of scale parameter. The starting point of all TTEs is ${d}_{ij}$ at the time of trial entry, and the hazard of a TTE is always constant, regardless of the time and number of recurrences (illustrated in Fig. 3a).

The second model uses the same Poisson process as the first one, but adopts the exponential distribution with different scale parameters between the subjects using random effect (i.e., inter-individual variability exists). It is referred to as the Mixed-Poisson process.

The third is the Weibull model, where the starting point of the first TTE is ${d}_{ij}$, as in the Poisson process, but the starting point of the second and subsequent TTEs is the time of the previous event (illustrated in Fig. 3b). Then, a Weibull distribution was assumed for the time between events within each subject. In addition to a scale parameter similar to an exponential distribution, the Weibull distribution contains the shape parameter. The Weibull distribution allows the hazard to vary with time depending on the setting of the shape parameter. As this model adopts a Weibull distribution with a common parameter from the first to the third TTE (i.e. the way the hazard changes are common from the first to the third TTE), we refer to it as the Weibull model (constant).

The fourth model uses the same Weibull model as the second one, but adopts the Weibull distribution with different parameters between the “first TTE” and the “second and third TTE” (i.e., the way the hazard changes is different between the first and second and third TTEs), and so it is referred to as the Weibull model (change).

In a simple RCT situation where an intervention effect exists, previous studies with time-independent covariates have shown that both the AG and PWP-TT models perform well for the Poisson process. On the other hand, it has been shown that only the PWP-TT model performs well for the Weibull model (constant), and only the AG model performs well for the Mixed-Poisson process [28].

To generate TTREs that can account for unidirectional switching, which is assumed to be estimating intervention effects using the CoxPH model and several extended CoxPH models, we use a data generation process for the CoxPH model with time-dependent covariates, based on the three event generation models previously described [29]. If the generated TTRE exceeds ${t}_{E}$ or ${t}_{F}$, it is treated as right-censored at ${t}_{E}$ or ${t}_{F}$.

In the generation of TTRE in the Poisson process and the Mixed-Poisson process, three pseudo-random numbers were generated independently from the uniform distribution $U(0, 1)$ and sorted in increasing order, ${u}_{1},\: {u}_{2},\: {u}_{3}$ in turn (${u}_{k},\: k=1,\: 2,\: 3$). If the scale parameter of the exponential distribution is $\lambda$, the baseline hazard function is $\lambda$, which is always constant regardless of the time or number of recurrences. The $k$ th TTRE of the $j$ th subject in the $i$ th cluster, when the starting point is not considered, is as follows:

$$\begin{aligned}{T}_{ijk}^{*}=\left\{\begin{array}{l}\frac{-\mathrm{log}({u}_{k})} {\lambda \mathrm{exp} ({\beta}^{\prime}x+{\tau }_{i}+{\tau}_{j})}\\ \qquad\qquad\mathrm{if}\:-\mathrm{log}({u}_{k})\:<\:\lambda \mathrm{exp} \left({\beta}^{\prime}x+{\tau}_{i}+{\tau}_{j}\right){w}_{ij},\\ \frac{[-\mathrm{log}\left({u}_{k}\right)-\lambda \mathrm{exp} \left({\beta }^{\prime}x+{\tau }_{i}+{\tau }_{j}\right){w}_{ij}+\lambda \mathrm{exp} ({\beta }^{\prime}x+{\beta }_{tik}+{\tau }_{i}+{\tau }_{j}){w}_{ij}]}{\lambda \mathrm{exp} ({\beta }^{\prime}x+{\beta }_{tik}+{\tau }_{i}+{\tau }_{j})} \\ \qquad\qquad\mathrm{if}\:-\mathrm{log}({u}_{k})\:\ge\: \lambda \mathrm{exp} \left({\beta }^{\prime}x+{\tau }_{i}+{\tau }_{j}\right){w}_{ij}\end{array}\right.,\end{aligned}$$

where ${\tau }_{i}$ and ${\tau }_{j}$ is the random effect on the variations between clusters and between subjects, ${\tau }_{i}\sim N\left(0, {\sigma }^{2}\right)$ and ${\tau }_{j}\sim N(0, {\sigma }_{s}^{2})$. ${\sigma }_{s}^{2}$ is 0 for the Poisson process and > 0 for the Mixed-Poisson process.

As already mentioned, ${\beta }_{tik}$ is the parameter of the intervention effect on the $k$ th recurrence of the $i$ th cluster, and ${w}_{ij}$ is the distance to switch for each subject from the trial entry. For simplicity, we omitted the ${\beta }^{^{\prime}}x$ for the time-independent covariates in the simulation. The TTRE, which is used in the analysis considering the starting point, is represented by ${T}_{ijk}={d}_{ij}+{T}_{ijk}^{*}$.

In the generation of TTRE in the Weibull model, three pseudorandom numbers were generated independently from the uniform distribution $U(0, 1)$, ${u}_{1},\: {u}_{2},\: {u}_{3}$ in the order in which they are generated (${u}_{k},\: k=1,\: 2,\: 3$). Let the scale parameter of the Weibull distribution for each recurrence be ${\lambda }_{k}$, and the shape parameter be ${\nu }_{k}$. The baseline hazard function is ${\lambda }_{k}{\nu }_{k}{t}^{{\nu }_{k}-1}$ and it is allowed to vary with time. The $k$ th TTRE of the $j$ th subject in the $i$ th cluster, when the starting point is not considered, is as follows:

$$\begin{aligned}{T}_{ijk}^{*}=\left\{\begin{array}{l}{\left(\frac{-\mathrm{log}({u}_{k})}{{\lambda }_{k}\mathrm{exp} ({\beta }^{\prime}x+{\tau }_{i})}\right)}^{{1}/{{\nu}_{k}}} \\\qquad\qquad \mathrm{if}\:-\mathrm{log}\left({u}_{k}\right)\:<\:{\lambda }_{k}\mathrm{exp} \left({\beta }^{\prime}x+{\tau }_{i}\right){{w}_{ijk}}^{{v}_{k}}\\ {\left(\frac{[-\mathrm{log}\left({u}_{k}\right)-{\lambda }_{k}\mathrm{exp} \left({\beta }^{\prime}x+{\tau }_{i}\right){{w}_{ijk}}^{{\nu}_{k}} +{\lambda }_{k}\mathrm{exp} ({\beta }_{tik}){{\mathrm{exp} \left({\beta }^{\prime}x+{\tau }_{i}\right)w}_{ijk}}^{{\nu}_{k}}]}{{\lambda }_{k}\mathrm{exp} ({\beta }_{tik})\mathrm{exp} \left({\beta }^{\prime}x+{\tau }_{i}\right)}\right)}^{{1}/{{\nu}_{k}}} \\\qquad\qquad\mathrm{if}\: -\mathrm{log}\left({u}_{k}\right)\:\ge\: {\lambda }_{k}\mathrm{exp} \left({\beta }^{^{\prime}}x+{\tau }_{i}\right){{w}_{ijk}}^{{v}_{k}}\end{array}\right..\end{aligned}$$

${\tau }_{i}$, ${\beta }_{tik}$, ${w}_{ijk}$, and ${\beta }^{^{\prime}}x$ were explained in the previous sentence. The TTRE that is actually used for the analysis considering the starting point is:

$$\left\{\begin{array}{cc}{T}_{ijk}={d}_{ij}+{T}_{ijk}^{*}& k=1\\ {T}_{ijk}={T}_{ijk-1}+{T}_{ijk}^{*}& k=2,\: 3\end{array}\right.$$

The parameters are ${\lambda }_{1}={\lambda }_{2}={\lambda }_{3}, {\nu }_{1}={\nu }_{2}={\nu }_{3}$ for the Weibull model (constant), and ${\lambda }_{1}\ne {\lambda }_{2}={\lambda }_{3}, {\nu }_{1}\ne {\nu }_{2}={\nu }_{3}$ for the Weibull model (change).

In the actual example, 31.4% of the residents died during the trial period. Therefore, in our simulation, we considered the time-to-terminal-event (TTTE) as independent of the distance to switch and TTRE. If the generated TTTE does not exceed ${t}_{E}$ or ${t}_{F}$ and it is before the third TTRE, it is treated as mid-trial right-side censoring at the occurrence of the terminal event. The scale parameter of the Weibull distribution for the terminal event is ${\lambda }_{c}$, and the shape parameter is ${\nu }_{c}$. Without considering the starting point, the TTTE of the $j$ th subject in the $i$ h cluster, ${C}_{ij}^{*}$, can be expressed using the probability density function as follows:

$$f\left(x\right)=\frac{{\nu }_{c}}{{\lambda }_{c}^{{\nu }_{c}}}{x}^{{\nu }_{c}-1} exp\left\{-{\left(\frac{x}{{\lambda }_{c}}\right)}^{{\nu }_{c}}\right\}, x>0$$

The TTTE used in the actual analysis considering the starting point is expressed as ${C}_{ij}={d}_{ij}+{C}_{ij}^{*}$.

### Parameter settings

The scale parameter for the exponential distribution in the generation of the TTRE by the Poisson process was set to $\lambda =0.003281$. This parameter was estimated based on the TTHA up to the third of the actual example, with all starting points set to zero. In addition, the inter-individual variability of the scale parameter in the generation of the TTRE by the Mixed-Poisson process was set to ${\sigma }_{s}^{2}=0.3455$. For this parameter, we used an estimate of the standard deviation of the normal distribution for the scale parameter based on the TTHA.

The scale and shape parameters of the Weibull distribution in the generation of TTRE using the Weibull model (constant) were set to ${\lambda }_{1}={\lambda }_{2}={\lambda }_{3}=0.004703,\: {\nu }_{1}={\nu }_{2}={\nu }_{3}=1.1219$. These parameters were estimated based on the TTHA, up to the third of the actual example. The starting point of the second and subsequent TTHA was the time of the previous hospitalisation.

The scale and shape parameters of the Weibull distribution in the generation of the TTRE using the Weibull model (change) were set to ${\lambda }_{1}=0.003599,\: {\lambda }_{2}={\lambda }_{3}=0.009910,\: {\nu }_{1}=1.5122,\: {\nu }_{2}={\nu }_{3}=0.9108$. These parameters were estimated based on the “first TTHA” and the “second and third TTHA” of the actual example, respectively. The starting point of the second and subsequent TTHAs was the time of the occurrence of the previous hospitalisation.

The scale and shape parameters of the Weibull distribution in the generation of TTTE as mid-trial right-side censoring were set to ${\lambda }_{c}=0.003674$ and ${\nu }_{c}=1.7191$. These parameters were estimated based on the time to death in the actual example.

Two parameters were set for the true intervention effect. The first is ${\beta }_{tik}={\beta }_{t}=-0.264$, which was calculated as $\mathrm{ln}(4.3/5.6)$ based on the secondary outcome of the actual example, number of hospitalisations per facility month. The second is ${\beta }_{tik}={\beta }_{t}=0$, a setting used in previous studies on event generation models: HR = 1, which indicates that there is no difference in the risk of event occurrence between the control and intervention conditions. In a simple RCT situation where there is no intervention effect, both the AG and PWP-TT models have been shown to perform well, regardless of the type of event generation model [28].

### Simulation set-up

For all simulations, we fixed ${t}_{S}=0$ at the beginning of the trial, ${t}_{E}=360$ at the end of the last step period, and the total sample size per simulation (total number of subjects per trial) $N=2000$. These settings were based on the fact that the actual example lasts for 12 months from the start of the trial to the end of the final step period; if one month is considered to be approximately 30 days, the trial period can be calculated as 12 × 30 = approximately 360 days, and the total number of subjects was 1700. Unless otherwise noted, the basic settings for each simulation scenario are as follows: the number of simulations is 1000, the event generation model consists of three types (Poisson process, Weibull model (constant), Weibull model (change)), the parameters of the true intervention effect are two ways ($-0.264,\: 0$), and $s\left(=m\right)=5,\: {n}_{i}=n=N/m=400,\: {W}_{d}=({t}_{E}-{t}_{S}) / (m+1)=60,\: {\sigma }^{2}=0,\: E=1,\: F=0$. The setting of $s=m=5$ is in reference to the fact that the number of steps in the actual example is five (Fig. 1).

Each simulation scenario is listed below. Scenario II applied two policies for each statistical model: (i) with stratification by clusters and (ii) without stratification by clusters. In all scenarios, except for scenario II, only (i) was applied.

In Scenario I, the number of steps (clusters) varied as $s\left(=m\right)=2, 4, 5, 8, 10, 20$ to investigate how the performance of each statistical model changed as the number of steps (clusters) increased. As the number of steps changes, it becomes $n=N/m=1000, 500, 400, 250, 200, 100,\: {W}_{d}=120, 72, 60, 40, 33, 17$. The results based on $s\left(=m\right)=5,\: n=400,\: {W}_{d}=60$ in this scenario were used as a reference throughout the simulations in our study.

In Scenario II, we varied the variance with respect to the random effect ${\tau }_{i}$, which represents the variation among clusters, as ${\sigma }^{2}=0.25,\: 0.5,\: 1$, and investigated how the performance of each statistical model changed as the variation between clusters increased.

In Scenario III, the follow-up period varied as follows, $F=1, 2, 3, 4$ to investigate how the performance of each statistical model changed as the follow-up period increased. The setting of $F$ is based on the follow-up period of 2.5 steps in the actual example (Fig. 1). In this scenario, the time point of the trial entry point was ${d}_{ij}={t}_{S}+(\left({t}_{F}-{t}_{S}\right)*e)/E$, and the subject was allowed to enter until the end of the follow-up period.

In Scenario IV, the follow-up period was changed to $F=1, 2, 3, 4$ to investigate how the performance of each statistical model changed as the follow-up period increased. In this scenario, the time point of the trial entry point was ${d}_{ij}={t}_{S}+(\left({t}_{E}-{t}_{S}\right)*e)/E$, and entry was terminated at the end of the final step period.

In Scenario V, we varied the timing of the trial entry as follows, $E=1.5,\: 2,\: 4,\: 6$ to investigate how the performance of each statistical model changed as trial entry was concentrated at an earlier stage of the trial.

In Scenario VI, the time of trial entry varied as follows, $E=1.5,\: 2,\: 4,\: 6$, and the follow-up period was changed to $F=1, 2, 3, 4$, to investigate how the performance of each statistical model changed in a situation where trial entry was concentrated in an earlier stage of the trial, and there was a follow-up period. In this scenario, for convenience, we used ${d}_{ij}={t}_{S}+(\left({t}_{E}-{t}_{S}\right)*e)/E$ as the time point for trial entry.

### Analysis of an actual example

The time-independent covariates employed in the model analysis for the primary outcome in the actual example (age, sex, medical power of attorney, health directive, advance care plan/statement of choices, primary diagnosis, age-adjusted Charlson comorbidity index, and fidelity) were used for adjustment, when analysing hospitalization > 24 h repeatedly occurred with the TTRE in the actual example using each statistical model.

Two policies were applied to each statistical model: (i) with stratification by clusters and (ii) without stratification by clusters. Fidelity is a per-cluster variable and was employed only with policy (ii), as it is not available for adjustment in (i). The unidirectional switch from the control condition to the intervention condition in each cluster was expressed using the intervention indicator as a time-dependent covariate.

In the usual TTRE analysis, continuous risk intervals were employed. However, in reality, they are not exposed to the risk of further hospitalisation during their hospital stay. Therefore, in this study, we adopted a discrete risk interval [30]. Thus, for example, if a resident was hospitalised, subsequent exposure to the risk of new hospitalisation would be from the day of discharge.

The results of the analysis were evaluated using HR and its 95% CI and *p*-value. In addition, parameter estimates and standard error (SE) were evaluated for the intervention effects.

### Software and code

All statistical analyses, including simulations, were performed using SAS, version 9.4 (SAS Institute, Cary, NC, USA). The PROC PHREG of SAS was used to analyse the TTRE. For the generation of pseudo-random numbers by SAS, the RANUNI function was used to generate the time point of trial entry and TTRE, the RANNOR function was used to generate the cluster effect, and the RAND function was used to generate the TTTE. For information on simulation codes, see Availability of data and materials.

## Results

### Simulation

The reference results for Scenario I with $s\hspace{1.5em}\left(=m\right)=5,\:n=400,\:W_d=60$ are shown in Table 1. These results were used as a reference for all the other simulations assessed in this study, as the setting $s \left(=m\right)=5$ references the fact that the number of steps in the actual example is five (Fig. 1).

**Table 1:** Performance for the reference results throughout the simulations

True Intervention effect (β_t_)	Event Generation model									
		AG model			PWP-TT model			PWP-GT model		
		Bias	MSE	CP	Bias	MSE	CP	Bias	MSE	CP
-0.264	Poisson	0.0266	0.0040	0.933	0.0021	0.0040	0.940	0.0380	0.0054	0.899
	Mixed-Poisson	0.0290	0.0041	0.927	0.0106	0.0039	0.938	0.0543	0.0067	0.844
	Weibull (constant)	0.0567	0.0067	0.858	0.0529	0.0065	0.864	0.0032	0.0037	0.949
	Weibull (change)	0.1247	0.0168	0.162	0.0328	0.0033	0.896	-0.0004	0.0022	0.955
0	Poisson	0.0023	0.0030	0.955	0.0024	0.0036	0.939	0.0021	0.0034	0.933
	Mixed-Poisson	0.0017	0.0028	0.958	0.0020	0.0034	0.941	0.0107	0.0033	0.936
	Weibull (constant)	0.0025	0.0032	0.948	0.0025	0.0034	0.948	0.0019	0.0031	0.944
	Weibull (change)	0.0011	0.0011	0.991	-0.0005	0.0020	0.961	-0.0011	0.0020	0.950

Settings: $$s\left(=m\right)=5,\: n=400,\: {W}_{d}=60,\: {\sigma }^{2}=0,\: E=1,\: F=0$$

From the reference results for ${\beta }_{t}=-0.264$, the MSE under the Poisson process and the Mixed-Poisson process was smaller for the AG and PWP-TT models, and slightly larger for the PWP-GT model; the CP performances of the AG and PWP-TT models were similar, but the bias was much smaller for the PWP-TT model. The PWP-GT model performed very well in both the Weibull model (constant) and Weibull model (change) but showed much lower performance in the Mixed-Poisson process. Under the Weibull model (change), the performance of the AG model was found to be very poor. In reference to the results for ${\beta }_{t}=0$, the overall performance was higher than that of ${\beta }_{t}=-0.264$. The AG model under the Weibull model (change) tended to overestimate CP. In all event generation models, the PWP-TT and PWP-GT models showed similar results, but the bias of the PWP-GT model in the Mixed-Poisson process was slightly larger than all other combinations.

The results for Scenario I when the parameter for the true intervention effect is ${\beta }_{t}=-0.264$ are shown in Table 2, and the results when ${\beta }_{t}=0$ are shown in Additional File (S.1). Regardless of the setting for ${\beta }_{t}$, the overall MSE increased slightly as the number of steps (clusters) increased, but this did not substantially impact on the performance comparison between the statistical models.

**Table 2:** Performance for scenario I with true intervention effect of ${\beta }_{t}=-0.264$

Event Generation model	Number of steps (clusters)									
		AG model			PWP-TT model			PWP-GT model		
		Bias	MSE	CP	Bias	MSE	CP	Bias	MSE	CP
Poisson	2	0.0267	0.0034	0.937	0.0006	0.0032	0.959	0.0393	0.0047	0.868
	4	0.0259	0.0036	0.941	0.0013	0.0036	0.948	0.0386	0.0051	0.894
	5	0.0266	0.0040	0.933	0.0021	0.0040	0.940	0.0380	0.0054	0.899
	8	0.0255	0.0041	0.943	0.0011	0.0041	0.938	0.0397	0.0056	0.896
	10	0.0251	0.0042	0.936	0.0016	0.0042	0.943	0.0376	0.0057	0.897
	20	0.0259	0.0042	0.938	0.0020	0.0042	0.949	0.0363	0.0057	0.912
Mixed-Poisson	2	0.0283	0.0035	0.930	0.0085	0.0032	0.947	0.0572	0.0065	0.785
	4	0.0284	0.0039	0.923	0.0099	0.0037	0.936	0.0563	0.0068	0.826
	5	0.0290	0.0041	0.927	0.0106	0.0039	0.938	0.0543	0.0067	0.844
	8	0.0282	0.0043	0.932	0.0097	0.0041	0.943	0.0547	0.0070	0.846
	10	0.0254	0.0042	0.937	0.0075	0.0042	0.947	0.0534	0.0071	0.845
	20	0.0281	0.0046	0.920	0.0097	0.0045	0.938	0.0530	0.0073	0.856
Weibull (constant)	2	0.0462	0.0052	0.870	0.0424	0.0050	0.882	0.0016	0.0032	0.944
	4	0.0546	0.0065	0.848	0.0509	0.0062	0.867	0.0003	0.0037	0.945
	5	0.0567	0.0067	0.858	0.0529	0.0065	0.864	0.0032	0.0037	0.949
	8	0.0563	0.0071	0.849	0.0522	0.0068	0.863	0.0016	0.0043	0.934
	10	0.0566	0.0072	0.842	0.0522	0.0069	0.860	0.0019	0.0042	0.945
	20	0.0553	0.0070	0.869	0.0496	0.0067	0.884	0.0004	0.0044	0.949
Weibull (change)	2	0.1229	0.0162	0.100	0.0253	0.0026	0.897	-0.0014	0.0019	0.950
	4	0.1221	0.0162	0.157	0.0299	0.0032	0.892	-0.0019	0.0021	0.963
	5	0.1247	0.0168	0.162	0.0328	0.0033	0.896	-0.0004	0.0022	0.955
	8	0.1240	0.0167	0.171	0.0330	0.0035	0.902	-0.0014	0.0024	0.954
	10	0.1250	0.0171	0.191	0.0348	0.0038	0.888	0.0010	0.0025	0.956
	20	0.1237	0.0169	0.222	0.0334	0.0038	0.901	-0.0012	0.0026	0.949

*AG* Andersen-Gill, *PWP-TT* Prentice-Williams-Peterson Total-Time, *PWP-GT* Prentice-Williams-Peterson Gap-Time, *MSE* Mean square error, *CP* Coverage probability

The results for Scenario II when the parameter for the true intervention effect is ${\beta }_{t}=-0.264$ are shown in Table 3, and the results when ${\beta }_{t}=0$ are shown in Additional File (S.2). Regardless of the setting of ${\beta }_{t}$, the performance of policy (ii) without stratification by clusters decreased as inter-cluster variation increased. At ${\sigma }^{2}=0.25$, the lowest variance in the setting, the decrease in performance was already apparent, especially for CP, as the performance was very poor. The reference results where policy (i) with stratification by clusters was performed in the absence of inter-cluster variation were similar to the results when (i) with stratification by clusters was performed in this scenario where inter-cluster variation was present.

**Table 3:** Performance for scenario II with true intervention effect of ${\beta }_{t}=-0.264$

	Event Generation model					
Dealing with clusters		Statistical model				
			σ^2^	Bias	MSE	CP
With stratification by clusters	Poisson process	AG	0.25	0.0250	0.0036	0.943
			0.5	0.0296	0.0040	0.933
			1	0.0358	0.0043	0.899
		PWP-TT	0.25	-0.0001	0.0037	0.957
			0.5	0.0016	0.0037	0.947
			1	0.0001	0.0036	0.951
		PWP-GT	0.25	0.0367	0.0051	0.898
			0.5	0.0387	0.0050	0.901
			1	0.0358	0.0047	0.892
	Mixed-Poisson process	AG	0.25	0.0296	0.0040	0.923
			0.5	0.0329	0.0043	0.917
			1	0.0397	0.0047	0.870
		PWP-TT	0.25	0.0105	0.0038	0.938
			0.5	0.0121	0.0039	0.941
			1	0.0143	0.0038	0.937
		PWP-GT	0.25	0.0545	0.0067	0.844
			0.5	0.0573	0.0071	0.820
			1	0.0591	0.0070	0.796
	Weibull model (parameter constant)	AG	0.25	0.0572	0.0069	0.844
			0.5	0.0556	0.0067	0.831
			1	0.0510	0.0061	0.836
		PWP-TT	0.25	0.0531	0.0066	0.852
			0.5	0.0512	0.0064	0.857
			1	0.0461	0.0058	0.861
		PWP-GT	0.25	0.0041	0.0038	0.950
			0.5	0.0015	0.0033	0.959
			1	0.0008	0.0033	0.939
	Weibull model (parameter change)	AG	0.25	0.1237	0.0166	0.158
			0.5	0.1196	0.0157	0.197
			1	0.1087	0.0134	0.275
		PWP-TT	0.25	0.0333	0.0034	0.892
			0.5	0.0323	0.0034	0.889
			1	0.0300	0.0033	0.886
		PWP-GT	0.25	-0.0002	0.0022	0.952
			0.5	-0.0006	0.0022	0.965
			1	0.0005	0.0022	0.954
Without stratification by clusters	Poisson process	AG	0.25	0.0362	0.0139	0.560
			0.5	0.0451	0.0438	0.324
			1	0.0666	0.1344	0.172
		PWP-TT	0.25	0.0057	0.0179	0.521
			0.5	0.0228	0.0537	0.289
			1	0.0634	0.1262	0.180
		PWP-GT	0.25	0.0361	0.0200	0.481
			0.5	0.0552	0.0595	0.274
			1	0.1067	0.1530	0.161
	Mixed-Poisson process	AG	0.25	0.0400	0.0139	0.556
			0.5	0.0472	0.0430	0.319
			1	0.0696	0.1286	0.179
		PWP-TT	0.25	0.0177	0.0162	0.534
			0.5	0.0318	0.0489	0.308
			1	0.0708	0.1159	0.177
		PWP-GT	0.25	0.0527	0.0196	0.477
			0.5	0.0697	0.0566	0.290
			1	0.1189	0.1440	0.166
	Weibull model (parameter constant)	AG	0.25	0.0417	0.0195	0.483
			0.5	0.0486	0.0623	0.270
			1	0.0720	0.1760	0.159
		PWP-TT	0.25	0.0405	0.0194	0.482
			0.5	0.0558	0.0567	0.286
			1	0.0950	0.1295	0.182
		PWP-GT	0.25	0.0085	0.0195	0.466
			0.5	0.0264	0.0606	0.260
			1	0.0828	0.1525	0.168
	Weibull model (parameter change)	AG	0.25	0.1227	0.0203	0.254
			0.5	0.1216	0.0338	0.276
			1	0.1227	0.0814	0.186
		PWP-TT	0.25	0.0281	0.0156	0.438
			0.5	0.0445	0.0456	0.258
			1	0.0835	0.1011	0.179
		PWP-GT	0.25	0.0068	0.0162	0.429
			0.5	0.0274	0.0506	0.255
			1	0.0845	0.1259	0.161

*AG* Andersen-Gill, *PWP-TT* Prentice-Williams-Peterson Total-Time, *PWP-GT* Prentice-Williams-Peterson Gap-Time, *MSE* Mean square error, *CP* Coverage probability

The results for Scenario III, when the parameter for the true intervention effect is ${\beta }_{t}=-0.264$ are shown in Table 4, and the results when ${\beta }_{t}=0$ are shown in Additional File (S.3). When ${\beta }_{t}=-0.264$, the performance of the AG and PWP-TT models under the Weibull model (constant) and the PWP-TT model under the Weibull model (change) improved as the follow-up period increased, when the trial entry was allowed until the end of the follow-up period. In particular, for CP, the performance was comparable to that of the PWP-GT model under the respective event generation model. On the other hand, the performance of the Mixed-Poisson process tended to be less than or equal to that of the reference results.

**Table 4:** Performance for scenario III with true intervention effect of ${\beta }_{t}=-0.264$

Event generation model										
		AG model			PWP-TT model			PWP-GT model		
	F	Bias	MSE	CP	Bias	MSE	CP	Bias	MSE	CP
Poisson	1	0.0307	0.0037	0.931	0.0015	0.0034	0.947	0.0408	0.0050	0.895
	2	0.0338	0.0038	0.936	0.0013	0.0034	0.947	0.0435	0.0051	0.878
	3	0.0358	0.0039	0.928	0.0013	0.0034	0.949	0.0447	0.0051	0.863
	4	0.0369	0.0041	0.929	0.0010	0.0036	0.952	0.0445	0.0052	0.873
Mixed-Poisson	1	0.0343	0.0041	0.919	0.0126	0.0036	0.945	0.0601	0.0070	0.804
	2	0.0366	0.0041	0.907	0.0125	0.0036	0.940	0.0612	0.0069	0.785
	3	0.0390	0.0044	0.911	0.0135	0.0037	0.940	0.0615	0.0070	0.790
	4	0.0405	0.0045	0.909	0.0139	0.0037	0.938	0.0624	0.0070	0.803
Weibull (constant)	1	0.0453	0.0052	0.874	0.0406	0.0049	0.894	0.0028	0.0030	0.953
	2	0.0377	0.0046	0.916	0.0326	0.0044	0.918	0.0011	0.0029	0.959
	3	0.0320	0.0042	0.932	0.0264	0.0041	0.934	0.0016	0.0030	0.940
	4	0.0280	0.0042	0.938	0.0219	0.0042	0.935	0.0022	0.0030	0.937
Weibull (change)	1	0.1227	0.0161	0.119	0.0235	0.0027	0.929	-0.0002	0.0020	0.951
	2	0.1206	0.0156	0.130	0.0173	0.0024	0.939	-0.0008	0.0020	0.944
	3	0.1203	0.0155	0.139	0.0148	0.0023	0.949	0.0001	0.0019	0.950
	4	0.1202	0.0155	0.133	0.0129	0.0024	0.953	0.0006	0.0020	0.948

*AG* Andersen-Gill, *PWP-TT* Prentice-Williams-Peterson Total-Time, *PWP-GT* Prentice-Williams-Peterson Gap-Time, *MSE* Mean square error, *CP* Coverage probability

The results for Scenario IV when the parameter for the true intervention effect was ${\beta }_{t}=-0.264$ are shown in Table 5, and the results when ${\beta }_{t}=0$ are shown in Additional File (S.4). When ${\beta }_{t}=-0.264$, the performance of the AG and PWP-TT models under the Weibull model (constant) and the PWP-TT model under the Weibull model (change), improved as the follow-up period increased, given the policy of terminating trial entry at the end of the final step period. However, none of them reached the same level of performance as the PWP-GT model in their respective event generation models. In contrast, the performance of the PWP-GT model under the Poisson process tended to decrease as the follow-up period increased. In addition, the performance of the Mixed-Poisson process tended to be less than or equal to that of the reference results.

**Table 5:** Performance for scenario IV with true intervention effect of ${\beta }_{t}=-0.264$

Event generation model										
		AG model			PWP-TT model			PWP-GT model		
	F	Bias	MSE	CP	Bias	MSE	CP	Bias	MSE	CP
Poisson	1	0.0320	0.0037	0.919	0.0020	0.0033	0.948	0.0429	0.0050	0.876
	2	0.0350	0.0036	0.911	0.0015	0.0031	0.944	0.0470	0.0052	0.840
	3	0.0361	0.0037	0.910	0.0012	0.0031	0.943	0.0497	0.0054	0.825
	4	0.0361	0.0037	0.909	0.0009	0.0031	0.944	0.0512	0.0055	0.810
Mixed-Poisson	1	0.0344	0.0038	0.921	0.0121	0.0033	0.941	0.0604	0.0066	0.799
	2	0.0377	0.0038	0.919	0.0128	0.0031	0.945	0.0639	0.0068	0.763
	3	0.0388	0.0038	0.911	0.0130	0.0030	0.945	0.0647	0.0068	0.754
	4	0.0388	0.0038	0.910	0.0127	0.0030	0.943	0.0643	0.0067	0.745
Weibull (constant)	1	0.0475	0.0051	0.866	0.0429	0.0048	0.882	0.0028	0.0028	0.948
	2	0.0418	0.0044	0.886	0.0367	0.0042	0.907	0.0024	0.0025	0.942
	3	0.0391	0.0042	0.890	0.0339	0.0039	0.912	0.0026	0.0023	0.945
	4	0.0381	0.0041	0.899	0.0329	0.0039	0.913	0.0022	0.0023	0.947
Weibull (change)	1	0.1282	0.0174	0.062	0.0264	0.0026	0.908	0.0001	0.0018	0.950
	2	0.1264	0.0169	0.065	0.0233	0.0024	0.922	-0.0002	0.0017	0.946
	3	0.1249	0.0165	0.068	0.0222	0.0023	0.930	-0.0004	0.0017	0.949
	4	0.1246	0.0165	0.069	0.0219	0.0023	0.930	-0.0004	0.0017	0.949

*AG* Andersen-Gill, *PWP-TT* Prentice-Williams-Peterson Total-Time, *PWP-GT* Prentice-Williams-Peterson Gap-Time, *MSE* Mean square error, *CP* Coverage probability

The results for Scenario V when the parameter for the true intervention effect is ${\beta }_{t}=-0.264$ are shown in Table 6, and the results when ${\beta }_{t}=0$ are shown in Additional File (S.5). Regardless of the setting of ${\beta }_{t}$, there was a tendency for the overall MSE to increase as the trial entry was more concentrated at the beginning of the trial. When ${\beta }_{t}=-0.264$, for the PWP-GT model under the Poisson process, the AG and PWP-TT models under the Weibull model (constant), and the PWP-TT model under the Weibull model (change), CP always performed poorly when compared to the reference results, regardless of the value for $E$. On the other hand, in the Mixed-Poisson process, Bias tended to decrease in the AG and PWP-TT models and increase in the PWP-GT model as the trial entry was more concentrated at the beginning of the trial.

**Table 6:** Performance for scenario V with true intervention effect of ${\beta }_{t}=-0.264$

Event generation model										
		AG model			PWP-TT model			PWP-GT model		
	E	Bias	MSE	CP	Bias	MSE	CP	Bias	MSE	CP
Poisson	1.5	0.0265	0.0040	0.937	0.0014	0.0041	0.937	0.0498	0.0064	0.848
	2	0.0232	0.0045	0.943	0.0015	0.0046	0.938	0.0603	0.0084	0.842
	4	0.0110	0.0088	0.944	-0.0012	0.0095	0.938	0.0786	0.0152	0.856
	6	0.0066	0.0119	0.954	-0.0021	0.0128	0.948	0.0878	0.0212	0.870
Mixed-Poisson	1.5	0.0289	0.0039	0.930	0.0098	0.0036	0.948	0.0660	0.0079	0.796
	2	0.0246	0.0045	0.944	0.0081	0.0045	0.945	0.0740	0.0096	0.809
	4	0.0144	0.0086	0.939	0.0050	0.0092	0.928	0.0869	0.0169	0.840
	6	0.0082	0.0119	0.947	0.0016	0.0124	0.945	0.0944	0.0218	0.863
Weibull (constant)	1.5	0.0721	0.0084	0.768	0.0689	0.0080	0.793	0.0002	0.0034	0.957
	2	0.0876	0.0115	0.724	0.0855	0.0112	0.734	0.0013	0.0042	0.945
	4	0.1148	0.0203	0.713	0.1139	0.0202	0.731	-0.0044	0.0080	0.944
	6	0.1274	0.0264	0.757	0.1267	0.0264	0.766	-0.0019	0.0107	0.957
Weibull (change)	1.5	0.1363	0.0201	0.122	0.0497	0.0050	0.834	-0.0005	0.0025	0.937
	2	0.1391	0.0214	0.213	0.0656	0.0073	0.780	0.0009	0.0031	0.954
	4	0.1437	0.0251	0.521	0.0961	0.0149	0.769	-0.0016	0.0063	0.949
	6	0.1447	0.0275	0.629	0.1061	0.0193	0.788	-0.0040	0.0089	0.952

*AG* Andersen-Gill, *PWP-TT* Prentice-Williams-Peterson Total-Time, *PWP-GT* Prentice-Williams-Peterson Gap-Time, *MSE* Mean square error, *CP* Coverage probability

The results for Scenario VI, when the parameter for the true intervention effect is ${\beta }_{t}=-0.264$, are shown in Additional File (S.6), and the results when ${\beta }_{t}=0$ are shown in Additional File (S.7), respectively. The results are similar to those of Scenario V, regardless of the setting of ${\beta }_{t}$ or the value of $F$.

### Actual example

The results summarising only the intervention indicators as time-dependent covariates are shown in Table 7. The overall results, including the time-independent covariates used for adjustment, are shown in Additional File S.8.

**Table 7:** Analysis results of actual example (intervention indicator only)

Dealing with clusters	Statistical model	Parameter Estimates	Standard Error		
				HR [95%CI]	*p*-value
With stratification by clusters	AG	0.033	0.122	1.034 [0.814, 1.314]	0.785
	PWP-TT	-0.061	0.123	0.941 [0.739, 1.198]	0.621
	PWP-GT	0.054	0.117	1.056 [0.840, 1.327]	0.641
Without stratification by clusters	AG	0.096	0.094	1.102 [0.915, 1.326]	0.306
	PWP-TT	0.040	0.095	1.041 [0.863, 1.254]	0.676
	PWP-GT	0.087	0.091	1.090 [0.912, 1.304]	0.360

*AG* Andersen-Gill, *PWP-TT* Prentice-Williams-Peterson Total-Time, *PWP-GT* Prentice-Williams-Peterson Gap-Time, *HR* Hazard ratio, *CI* Confidence interval

The HR for the intervention indicator shows the relative risk of the intervention condition when compared to the control. Except for the PWP-TT model under policy (i) with stratification by clusters, the overall HR was slightly above 1, suggesting that the risk of events in the intervention condition may be higher than in the control, although the difference was not statistically significant. Reviewing the results of the statistical model, under policy (ii) without stratification by clusters, the HR tended to be larger, and the range of the SE and 95% CI was smaller than under policy (i) with stratification by clusters.

The results of the covariates other than the intervention indicator, showed that the primary diagnosis of “dementia and Parkinson’s disease”, and the age-adjusted Charlson comorbidity index were statistically significant for all statistical models. Residents with dementia and Parkinson’s disease had a lower risk of event occurrence than those without dementia and Parkinson’s disease, suggesting that the risk of event occurrence may increase with the severity of comorbidities.

## Discussion

In this study, we have conducted comparative simulations to identify the statistical model’s whose performance for estimating intervention effects based on TTRE in SWCRT using an open cohort design were superior and could effectively be applied to actual clinical trial data.

The results of the simulations show that the performance under policy (ii) without stratification by clusters was worse when compared with policy (i) with stratification by clusters, in both the statistical models and settings. As SWCRT is implemented at the cluster level, it is necessary to consider that “cluster effects may exist” in any situation. Furthermore, even if there is no variation among the clusters, there is no difference in performance with and without stratification by clusters, so (i) with stratification by clusters should always be adopted in the estimation of intervention effects based on TTRE in SWCRT when using an open cohort design.

The results of the simulations, in a situation where there is no follow-up period, and the timing of the trial entry tends to be random, showed that Poisson processes were similar to those of previous studies in settings that did not include time-dependent covariates [28]. We found that the performance of the PWP-TT model decreases for the Weibull model (constant) and increases for the Mixed-Poisson process, which is somewhat different from previous studies. This is a tendency that is considered to be specific to SWCRT with an open cohort design.

In real-world SWCRT, there may be situations in which a follow-up period is established, or trial entry is concentrated in the early period, due to the nature of the study objectives and target clusters. The simulation results are important because they show that the performance of the statistical models against TTRE depends not only on the true intervention effects and event generation model, but also on the trial design of SWCRT (the presence of a follow-up period and the timing of trial entry).

The Mixed-Poisson process is an event-generating model that induces inter-individual variability in the Poisson process. Overall, in simulations in the presence of intervention effects, the bias in all statistical models was positively larger in the Mixed-Poisson process than the Poisson process; that is, it tended to underestimate the intervention effects. This is a similar result to simulations in previous studies [22]. In addition, the simulation as a whole tended to significantly degrade the performance of PWP-GT, especially in the Mixed-Poisson process with inter-individual variability, compared to the Poisson process and Weibull model without inter-individual variability. Since PWP-GT is the only one that is assumed to be “gap-time independent”, the result that the Mixed-Poisson process with no gap-time independence degrades performance is natural.

The event generation model used in our study was only a simulation assumption. The primary analysis methods in the clinical trials usually need to be specified in advance in the study protocol or statistical analysis plan. If the policy is to adopt a statistical model for the primary analysis, and it needs to determine a statistical model in the early phase of trial planning, it would be desirable to adopt one that shows reasonable performance in various settings, rather than one that performs well only in a particular event generation model. In our study, through simulations based on various settings, the PWP-GT model with stratification by clusters showed the best performance in most settings and reasonable performance in other settings, in situations where inter-individual variability did not exist. On the other hand, the PWP-GT model with stratification by clusters consistently underperformed compared to the PWP-TT model with stratification by clusters, in situations where inter-individual variability existed. Therefore, if the policy is to adopt a statistical model as the primary analysis, and this needs to be determined in the early phase of the trial planning, the PWP-TT model with stratification by clusters should be adopted if the inter-individual variability is known to be high from previous studies, and the PWP-GT model with stratification by clusters should be adopted if it is not.

Under the Weibull model (change), the overall performance of the AG model tended to be very low when intervention effects were present, and the CP of the AG model tended to be excessive when there were no intervention effects. The AG model assumes a common baseline hazard function for all events, independent of the number of previous recurrences. In contrast, in the Weibull model (change), the hazard clearly changes between the first event and the second and subsequent events. Therefore, it is to be expected that the performance of the AG model degrades under the Weibull model (change), theoretically. However, we believe that further research is needed on the cause of the terrifically low performance of CP. Anyway, considering the possibility that the actual event generation model is a Weibull model (change), it is challenging to adopt the AG model during the early phase of trial planning.

Under conditions where inter-individual variability does not exist, the only situation in which the performance of the PWP-TT model with stratification by clusters is not inferior to that of the PWP-GT model with stratification by clusters is when there is a certain amount of follow-up period, and the timing of the trial entry tends to be random within the trial period, including the follow-up period. Therefore, in this situation, it may be acceptable to adopt the PWP-TT model with stratification by clusters during the early phase of the trial planning, instead of the PWP-GT model with stratification by clusters. In our study, the performance of the PWP-TT model was particularly good when the follow-up period was more than three steps. In addition, considering that the original trial period consisted of six steps ($s=m=5$), it may be possible to think of it as a rough guide that “a certain amount of follow-up period” as “a follow-up period that is more than half the length of the original trial period”. The results presented in Additional File (S.9) indicate that it can be assumed that the same is true for different numbers of steps (clusters). The choice of which statistical model to use depends on the nature of the intervention, the characteristics of the subjects, and the clinical interpretability of the analysis results. In our study, for the sake of comparability, we estimated only the overall effects based on the PWP model, assuming that each recurrence had a common effect. However, in an actual analysis, it is possible to estimate event-specific effects. The PWP-TT model is appropriate when one wants to know the effect of each recurrence since the start of the subject’s follow-up. On the other hand, the PWP-GT model is suitable for understanding the effect of recurrence, in relation to the previous occurrence.

A previous study on the CoxPH model in the context of SWCRT showed a tendency for the MSE to decrease as the number of steps (clusters) increased. However, the simulations in our study showed an opposite trend. This difference is not apparent, but it is thought to be due to the differences in the various settings during the simulation. For example, in the previous study, the true intervention effect was set to 1, whereas in our study, it was set to − 0.264 or 0.

The observed numbers of recurrence per subject and the censoring proportions will be different depending on the scenario. Also, a previous study that evaluated the performance of the CoxPH model in SWCRT mentioned that the control-to-intervention ratio (the ratio of the total time in the control condition to the total time in the intervention condition) is related to the estimation accuracy [15]. A summary of this information for each scenario is given in Additional file (S.10) when the true intervention effect parameter is ${\beta }_{t}=-0.264$, and a summary for ${\beta }_{t}=0$ is given in Additional file (S.11). It may be helpful to take this information into account to interpret the simulations’ results for each scenario.

There is a follow-up period in the actual example, and trial entry is concentrated early in the trial period. Therefore, based on the results of the simulations, if the policy is to adopt a statistical model for estimating intervention effects based on TTRE against the actual example as the primary analysis, and this needs to be determined in the early phase of trial planning, the PWP-TT model with stratification by clusters should be adopted if the inter-individual variability is known to be high from previous studies, and the PWP-GT model with stratification by clusters should be adopted if it is not. However, considering that the parameter estimates are close to zero for any statistical models, if one model is adopted as the primary analysis, the other might be adopted as the exploratory analysis. The number of hospitalizations per facility-month, was evaluated as a secondary outcome in the actual example and showed an obvious decrease in the intervention condition when compared to the control, which is a substantial deviation from the results from the TTRE analysis of our study. One possible reason for this is that the analysis of the number of hospitalizations per facility-month ignores that residents are exposed to both the control and intervention conditions. The purpose of our study was to provide a different perspective to the existing evaluations. Therefore, it does not negate the conclusions of the actual example, which have previously been published.

Our study had several limitations. First, all of the statistical models employed treat a terminal event before the third TTRE as a mid-trial censoring event. However, if a death occurs, for instance, in actual example, the possibility of a subsequent hospitalisation is lost. An event such as a death in such a situation is called a competing risk [31], but in our study, we did not account for terminal events as competing risks.

Second, we assumed non-informative censoring for the terminal event, which was treated as mid-trial censoring. This assumes that censoring occurs independently due to causes unrelated to the TTRE. However, if, for example, repeated hospitalisations occur in an actual example, the risk of death is likely to increase. In such situations, it is possible to use an approach that considers the terminal event as informative censoring and corrects for it, but this was not applied [32, 33].

Third, the simulation in our study employed continuous risk intervals as it has been adopted in many previous studies [19, 22, 34]. However, we believe that simulations for discontinuous risk intervals (adopted in the analysis of the data from actual example) should be considered in the future.

Fourth, for simulation simplicity, we assumed that the number of clusters moving from the control condition to the intervention condition in one step was one ($s=m$). However, in actual example, two or three care homes are included in one cluster that transitions in one step. If the intervention effects can be assumed to be common among multiple care homes within a cluster, this is not an issue. If they cannot, they should be considered in the analysis, but we were not able to do this in our study.

Fifth, we adopted only “stratification by clusters” to handle the cluster effect. In our study, we were more interested in the differences in performance due to the differences in the design of SWCRT itself rather than the differences in performance due to the way the cluster effect is handled. In a previous study that evaluated the performance of the CoxPH model in SWCRT, both “stratified by cluster” and “frailty” were used for the cluster effect, and no difference in performance was found [15]. Based on the results, the “frailty” method, which is computationally expensive and takes a lot of time, was not included in our study from the beginning. However, stratification does not allow us to infer the effects of cluster-level variables (such as “fidelity” in actual example). Also, frailty is more appropriate when the clusters we are dealing with are considered to be drawn from a larger population of clusters. Therefore, examination using frailty is an issue that needs to be addressed in the future.

## Conclusions

Our objective was to evaluate which of the AG, PWP-TT, and PWP-GT models performed best in estimating the intervention effects using TTRE in SWCRT with an open cohort design. The performance was evaluated by Bias, MSE, and CP based on different event generation models and true intervention effects and several scenarios involving the SWCRT design itself. The simulation results showed that the PWP-GT model with stratification by clusters showed the most reasonable performance in situations where inter-individual variability was not present, especially when evaluated by CP, regardless of the presence of cluster effects. However, if inter-individual variability was present, the PWP-TT model with stratification by clusters performed best.

## Supplementary Information

**Additional file 1.**

## Data Availability

Simulation codes supporting the conclusions of this article are available from a GitHub repository at https://github.com/s-oyamada/TimeToRecurrentEventInSteppedWedge.

## Abbreviations

RCT: Randomized controlled trial
CRT: Cluster randomized trial
SWCRT: Stepped wedge cluster randomized trial
TTHA: Time-to-hospital admission
TTE: Time-to-event
TTFE: Time-to-first-event
TTRE: Time-to-recurrent-event
TTTE: Time-to-terminal-event
CoxPH: Cox proportional hazard
AG: Andersen-gill
PWP-TT: Prentice-williams-peterson total-time
PWP-GT: Prentice-williams-peterson gap-time
HR: Hazard ratio
MSE: Mean square error
CP: Coverage probability
CI: Confidence interval
SE: Standard error
